# Recognition of Familiar Wordforms and Phonological Variation in Akan by Multilingual Infants Learning African Tone Languages in Ghana

**DOI:** 10.1111/desc.70253

**Published:** 2026-07-07

**Authors:** Paul Okyere Omane, Reginald Akuoko Duah, Thierry Nazzi

**Affiliations:** ^1^ Language and Cognition Team, Integrative Neuroscience and Cognition Center CNRS–Université Paris Cité Paris France; ^2^ Department of Linguistics University of Ghana Legon Ghana

**Keywords:** African tone languages, Akan, language acquisition, multilingualism, phonological developments, wordform recognition

## Abstract

Recent developmental evidence shows that the proposal that consonants are more important than vowels for lexical processing is not universal but language‐specific. In non‐tone languages, a consonant bias emerges early in development in Romance languages (e.g., French) but later in Germanic languages (e.g., English), while evidence from Asian tone languages (Mandarin, Cantonese) shows a reverse vowel bias. The V‐bias in tone languages has been linked to the presence of lexical tones—mainly carried by vowels—which may increase vowel prominence. The present study tests the hypothesis that a V‐bias is more generally found in tone languages by examining infants learning an understudied African tone language, Akan (Kwa, Niger‐Congo). More specifically, we tested Akan‐learning multilingual infants (11–14 months) in Ghana (Sub‐Saharan Africa) in a wordform recognition task, using a central fixation procedure. After establishing that these infants preferred familiar Akan words over pseudowords (Experiment 1), Experiment 2 assessed whether one‐feature consonant or vowel mispronunciations of these words would differentially block recognition, indicative of a V‐bias. Infants showed comparable sensitivity to consonant and vowel mispronunciations, failing to establish a bias in these infants. Both findings were not modulated by age and relative exposure to Akan (and also relative exposure to tone languages in Experiment 2). Our findings add to existing evidence that early lexical processing is language‐specific. They also suggest that the emergence of a V‐bias in tone languages may depend on tone complexity and developmental age.

## Summary

- Akan‐learning multilingual infants in Ghana can recognize familiar wordforms.
- Akan‐learning multilingual infants show equal sensitivity to consonant and vowel mispronunciations of familiar wordforms.
- Minimal exposure to Akan may be sufficient for multilingual infants to recognize native language familiar wordforms.
- Exposure to African tone language(s) might not lead to a vowel bias, but might still reduce the often observed consonant bias in lexical processing.

## 1 Introduction

Most of what we know about early lexical acquisition and processing is based on studies of infants learning Indo‐European languages and growing up in Europe and North America; research on other languages and populations is rare. This lack of diversity and inclusivity has serious implications for language acquisition theories and their generalizability (e.g., Aravena‐Bravo et al. 2023; Cristia et al. 2023; Kidd and Garcia 2022; Scaff et al. 2025; Singh et al. 2023). Early language experience varies significantly across infants in different aspects, including the linguistic properties of their native languages, the linguistic context (e.g., multilingualism) of their environment, cultural differences, and the socialization contexts in which infants are raised. Therefore, only by adopting a more inclusive and diverse research approach can we better understand the language‐general and language‐specific factors that shape language acquisition and its developmental trajectories. In line with this, the present study focuses on infants growing up in Accra, Ghana, Sub‐Saharan Africa (an understudied population), learning several understudied languages (Omane, Benders, et al. 2025). More specifically, the present study investigated early wordform recognition and the link between phonological and lexical processing in Akan (a Kwa, Niger‐Congo language) by these multilingual infants. This is the first study to investigate the relative weighting of segmental features (i.e., consonants and vowels) in lexical processing in infants growing up in Africa, an issue that has received sustained attention in recent years.

This work relates to the proposal that consonants and vowels play distinct roles in language acquisition, leading to phonological biases in early language acquisition and processing, as described by the division of labor hypothesis (Nespor et al. 2003). This hypothesis argues that consonantal information is most relevant for lexical acquisition and processing, while vowel information is most relevant for prosodic and syntactic development. Thus, with respect to the relative weighting of consonants and vowels in lexical acquisition and processing, language learners and adults alike are predicted to rely more heavily on consonantal information, a bias referred to as the consonantal bias (C‐bias).

In adults, the literature supporting the C‐bias primarily comes from studies on several Indo‐European languages (such as English or French), although evidence was also found in Sesotho, Japanese, and Hebrew (see Nazzi and Cutler 2019). For example, in a word reconstruction task, English‐, Dutch‐, and Spanish‐speaking adults were presented with nonwords and asked to transform them into real words by changing a single sound. Results revealed that all language groups showed a stronger tendency to preserve consonantal than vocalic information and were significantly faster and more accurate when changing vowels than when changing consonants (van Ooijen 1996; Cutler et al. 2000). In contrast, results on two Asian tone languages, Mandarin and Cantonese, failed to find a C‐bias (e.g., Wiener and Turnbull 2016; Gómez et al. 2018; Poltrock et al. 2018; Wiener 2020). For Mandarin, in a word reconstruction study, Mandarin‐speaking adults preferred to modify tones rather than vowels or consonants for which there was no difference, indicating no C‐bias (Wiener and Turnbull 2016). Yet, a follow‐up study with more controlled stimuli found a clear V‐bias in Mandarin‐speaking adults (Wiener 2020). For Cantonese, an eye‐tracking study investigating how Cantonese‐speaking adults process consonants, vowels, and tones when learning new words in their native language showed no difference in performance across the three conditions, providing no evidence of a C‐bias (Poltrock et al. 2018). Yet, a wordform segmentation study found that Cantonese‐speaking adults can learn new words in an artificial language based on the combination of its vowels and tones, but not on its consonants, providing some evidence of a V‐bias (Gómez et al. 2018). Taken together, these adult studies indicate that the C‐bias is not a universal bias in lexical processing but is instead cross‐linguistically modulated. This suggests that adults weigh consonantal and vocalic information differently across languages, likely as a result of the properties of their native language. It has been argued that the presence of lexical tones, which contrast word meaning and thus play a critical role in lexical processing and identification in tone languages, may alter the relative weighting of consonants and vowels. Because tones are pitch variations carried by vowels, their presence increases vowel variability in tone languages compared to non‐tone languages. Moreover, because tones serve a lexical function, speakers of tone languages might attend more closely to vowels by paying more attention to tones. Both elements should increase the relative weight and perceptual prominence of vowels compared to consonants, potentially attenuating or reversing the C‐bias in tone languages.

Since language‐specific biases develop early in infancy, several studies have explored the origin of the C‐bias in development and whether it is modulated cross‐linguistically, testing two broad accounts. On the one hand, the C‐bias has been proposed to be innate: infants are assumed to process consonants and vowels differently from birth, and this bias is argued to be universal and independent of the specific properties of the native language (Nespor et al. 2003). On the other hand, the C‐bias might emerge from the linguistic properties of infants’ ambient language(s). The acoustic/phonetic hypothesis argues that inherent acoustic/phonetic differences between consonants and vowels (such as consonants being typically shorter, less periodic, and perceived more categorically than vowels) guide phonological category formation, leading to the emergence of the C‐bias during the first year of life (Floccia et al. 2014). In parallel, the lexical bias hypothesis proposes that the C‐bias arises from experience with the developing lexicon: once infants acquire a sufficiently large vocabulary, around 12 months, they learn that consonants provide more informative cues to lexical identity than vowels (Keidel et al. 2007). More recently, the phono‐lexical hypothesis argues that phonological and lexical properties jointly contribute to the emergence of the C‐bias, viewing the acoustic/phonetic and lexical influences as complementary rather than mutually exclusive (Poltrock and Nazzi 2015).

Many studies have explored the consonant bias in lexical acquisition and processing in infancy and toddlerhood across languages (e.g., French, Italian, Spanish, English, German, Mandarin, and Cantonese), using a range of experimental procedures such as word segmentation, word learning, and word(‐form) recognition (see Nazzi and Cutler 2019; Nazzi 2025, for reviews). Studies of Romance languages report an early emergence of the C‐bias, from around 8 months in French (e.g., Nishibayashi and Nazzi 2016; Nazzi 2005; Poltrock and Nazzi 2015; Von Holzen and Nazzi 2020), Italian (Hochmann et al. 2011), and Spanish (Bouchon et al. 2022). Before this emergence, a V‐bias is found in French‐learning infants aged 5–8 months (Bouchon et al. 2015; Nishibayashi and Nazzi 2016; Von Holzen and Nazzi 2020), in Italian newborns (Benavides‐Varela et al. 2012), and in Spanish‐learning infants at 6 months (Hochmann et al. 2018). In contrast, studies of Germanic languages report a later emergence of the C‐bias. British English–learning toddlers show evidence of a C‐bias at 30 months (Nazzi et al. 2009), but not at 11 months (Ratnage et al. 2024); 16 or 23 months (Floccia et al. 2014); or 12, 18, or 24 months, although a weak C‐bias was reported at 15 months (Mani and Plunkett 2007, 2010). Danish‐learning 20‐month‐olds show either a V‐bias (Højen and Nazzi 2016) or no bias (Højen et al. 2023), while no bias was found in German‐learning 24‐month‐olds (Piot et al. 2025). Overall, these findings indicate that the C‐bias is not present from birth and is acquired during the first years of life, with the timing of its emergence being modulated across languages.

Different from the developmental trajectories observed in the Romance and Germanic languages, studies of Mandarin and Cantonese reveal a different pattern, with no evidence of a C‐bias across infancy and toddlerhood (see Nazzi 2025, for a review). The first study to investigate the C‐bias in Mandarin tested Mandarin–English bilingual toddlers (2.5–3.5 years) and preschoolers (4–5 years), assessing their sensitivity to correctly pronounced familiar words and to mispronunciations of these words involving consonant, vowel, or tone changes (Singh et al. 2015). Toddlers were more sensitive to tone mispronunciations than to consonant or vowel mispronunciations, whereas preschoolers showed the opposite pattern. No difference was found between consonant and vowel mispronunciations in either age group, showing no C‐bias in Mandarin. A follow‐up study found no difference between consonant, vowel, and tone mispronunciations in Mandarin monolinguals and Mandarin‐English bilinguals, showing no C‐bias; however, analyses of response times revealed a V‐bias at 24 months in both groups (Wewalaarachchi et al. 2017). Last, neither a C‐ nor a V‐bias was found in Mandarin monolinguals and Mandarin–English bilinguals at 6 years (Wewalaarachchi and Singh 2020). In Cantonese, a word learning study tested whether Cantonese‐learning toddlers aged 20 and 30 months can learn minimally contrasting word pairs differing in a consonant, a vowel, or a tone, to assess the relative weighting of these contrasts during word learning (Chen et al. 2021). No word learning and no bias were found at 20 months; however, at 30 months, toddlers could learn in the vowel contrasted condition only (with a marginal effect in the tone contrasted condition), showing a V‐bias. The evidence from Asian tone languages suggests that a C‐bias is absent in tone languages and that the presence of tones might lead to a V‐bias, in line with the adult results reviewed above. Taken together, the developmental evidence so far establishes that the C‐bias in lexical acquisition and processing is language‐specific rather than universal, and acquired early in life rather than being innate.

Given that current evidence on tone languages comes primarily from Asian tone languages with similar tonal systems but varying levels of complexity, more research is needed to determine whether a V‐bias is consistent across tone languages. A further limitation of the research on the C‐bias is that, to date, no study has examined the C‐bias in multilingual infants, nor how early multilingual exposure influences its development. Although some published studies have explored the C‐bias in bilingual infants and toddlers (Singh et al. 2015; Wewalaarachchi and Singh 2020), none have examined how bilinguals’ exposure (e.g., their relative exposure to each of their languages) affects their lexical processing and the development of a bias.

The present study extends previous work to an understudied African tone language (Akan, Kwa, Niger‐Congo) and multilingual infants learning Akan alongside other local tone languages (e.g., Ga, Ewe) and a non‐tone language (Ghanaian English). It investigates whether a V‐bias extends beyond Asian tone languages. The tonal system of Akan consists of two contrastive tones, high (H) and low (L), which serve both lexical and grammatical functions (see Dolphyne 1988). It differs from those of Mandarin and Cantonese in two ways. In terms of complexity, Akan has fewer tones than Mandarin (4) and Cantonese (6). Typologically, Akan is a register‐tone language, while Mandarin and Cantonese are contour‐tone languages, with Cantonese also having register tones. Hence, besides broadening the scope of the tone languages investigated, the present study will also bring new data on how complexity and typological differences may influence the relative weighting of consonants and vowels in learners of tone languages, as suggested by Chen et al. (2021) in their discussion of why stronger V‐bias effects seem to be found in Cantonese than in Mandarin.

The present study will thus investigate whether Akan‐learning multilingual infants have a V‐bias in lexical processing in Akan. It will test them at 11 months, on their sensitivity to consonant and vowel mispronunciations in wordform recognition. However, to be able to examine the relationship between wordform recognition and phonological processing in these infants, we first conducted a baseline study to determine whether infants can recognize familiar Akan words.

### 1.1 Experiment 1–Familiar Wordforms Versus Pseudowords

This experiment investigated the recognition of correctly pronounced familiar Akan words by Akan‐learning multilingual infants aged 11–14 months. Based on previous findings on familiar wordform recognition (e.g., Hallé and de Boysson‐Bardies 1994; Vihman et al. 2004; Swingley 2005; Poltrock and Nazzi 2015), we hypothesized that Akan‐learning multilingual infants would show a preference for familiar Akan wordforms over pseudowords, indicating early recognition of familiar Akan words. We also investigated whether percentage of exposure to Akan modulates this preference, predicting a larger preference with increased exposure.

## 2 Method

### 2.1 Participants

Twenty‐four 11‐ to 14‐month‐old infants (9 girls and 15 boys, *M*_age_ = 393 days; range = 334–451 days) learning Akan and other languages were tested and included in this study. This target sample size was determined based on previous word recognition studies, in which 12–24 infants are commonly tested (e.g., Hallé and de Boysson‐Bardies 1994; Poltrock and Nazzi 2015). While we acknowledge that this limited sample may raise potential power concerns, given that this was fieldwork with a limited stay period, 24 infants was the realistic maximum sample we could reach. An additional six infants were tested but excluded from the analyses due to the child crying (*n* = 3) or parental interference (*n* = 3). All infants were Africans, born in Ghana, raised multilingually, recruited and tested in urban Accra (Ghana). Recruitment occurred through three primary mediums: (1) schools (e.g., crèches); (2) antenatal centers; and (3) snowball sampling through personal networks. To obtain a more diverse sample, infants were recruited from multiple communities within Accra. Eligibility for participation required exposure to at least Akan. A parental questionnaire was used to assess the infants’ linguistic backgrounds (see Section 2.5). The data showed that infants were exposed to two to five languages (4.2% two languages, 79.1% three languages, 12.5% four languages, and 4.2% five languages; see Section 4 for more details). Regarding socioeconomic status, maternal education data showed that all parents or caregivers had some formal education, ranging from junior high school (9 years of education) to a bachelor's or polytechnic degree (16 years of education). No infant was reported to have developmental disabilities or delays. Caregivers received a fee as compensation for their participation. We followed the guidelines in the Declaration of Helsinki and obtained informed written consent from parents or caregivers prior to participation. The study was approved by the Ethics Committee for Humanities at the University of Ghana (Ghana). Participants received a fee as compensation for their participation.

### 2.2 Task Design

Our experimental design closely followed previous wordform recognition studies. The composition of familiar word and pseudoword trials, as well as the number of test trials per infant, was based on Poltrock and Nazzi (2015). However, unlike Poltrock and Nazzi (2015), we used the single‐screen central fixation procedure (Cooper and Aslin 1990).

### 2.3 Auditory Stimuli

Ten familiar bi‐syllabic CVCV (C = Consonant, V = Vowel) Akan words and bi‐syllabic pseudowords were used in this experiment (see Table 1). The familiar Akan words were selected from data obtained in two prior studies that collected Akan CDI or checklist information (Coffey et al. 2025; Prado et al. 2016). The pseudowords were created in two ways following the phonological properties of Akan: (1) by combining syllables from different familiar words (e.g., pafɪ) and (2) by combining a CV syllable from a familiar word with CV syllable not present in any familiar words (e.g., dɛwɔ) to avoid creating real words, while ensuring that consonants and vowels used in both words and pseudowords were matched as closely as possible.

**TABLE 1:** List of familiar words and pseudowords used in Experiment 1.

Familiar Akan words	Pseudowords
tɔ̀fɪ́	pàfɪ́
kʷæ̀dú	fàrɪ́
sɔ̀rɪ́	dùrí
dʑìnà	fìdʑà
wɔ̀fà	dɛ̀wɔ̀
dʑɥàrɪ̀	wùhɔ̀
pìrà	kìnà
hùrì	dæ̀sì
nípá	bíná
sɪ̀wá	rìnú

*Note*: Transcription of words is based on the IPA.

The stimuli were recorded by a female native Akan speaker in an infant‐directed manner. The recording took place in a sound‐attenuating booth at the Université Paris Cité, using a Tascam DR‐O7 MKII recorder. Further processing of the stimuli (e.g., extraction of words and pseudowords, checking the intensity and duration of the words) was done using Praat Software (version 6.3.10, Boersma and Weenink 2023). Following Poltrock and Nazzi (2015), we selected three tokens of each item and constructed six pseudo‐randomized lists for each condition (six “familiar word lists” and six “pseudoword lists”). In each list, the 10 familiar words or pseudowords were presented twice (each in the first half and the second half of the list), using different tokens of each item in the two halves. This yielded a total of 20 tokens per list. The order of the items was pseudo‐randomized, ensuring that the presentation order of each familiar word and pseudoword was well distributed within and across the lists. All lists were 23.00 s in duration, with an interstimulus interval ranging from 609 to 617 ms. Acoustic analyses were performed on word duration, intensity, and fundamental frequency measures (mean, minimum, and maximum F0) to verify the lack of differences between the familiar and pseudoword lists that could have influenced looking times (Table 2).

**TABLE 2:** Acoustic parameters of the familiar versus pseudowords (means [SDs], *t*‐ and *p*‐values).

	Conditions		Test statistics
	Familiar word	Pseudowords	
Duration (seconds)	568 (60.9)	567 (53.0)	*t* (58) = 0.06, *p* = 0.96
Intensity (db)	69.9 (2.4)	69.8 (3.2)	*t* (58) = 0.12, *p* = 0.91
Minimum F0 (Hz)	100.8 (13.4)	104.8 (17.6)	*t* (58) = –0.99, *p* = 0.33
Maximum F0 (Hz)	169.6 (28.7)	175.3 (18.5)	*t* (58) = –0.91, *p* = 0.37
Averages (Hz)	136.1 (18.5)	137.3 (14.3)	*t* (58) = –0.28, *p* = 0.78

### 2.4 Visual Stimuli

Two visual stimuli were used in the experiment: a colorful spinning wheel, which served as an attention‐getter before the auditory trial started, and a static checkerboard, which served as an unrelated visual stimulus, presented simultaneously with the auditory stimulus during the test trials.

### 2.5 Procedure

#### 2.5.1 Experimental

The single‐screen central fixation procedure (Cooper and Aslin 1990) was used. The experiment was programmed and run using E‐prime 3.0 on a Dell Latitude 7420 foldable laptop. Each infant was tested individually in a quiet room. Both the experimenter and the caregiver listened to music over noise‐canceling headphones, masking the auditory stimuli. To minimize the potential influence of the experimenter's activities (e.g., coding and switching trials) during the experimental session, the testing room was partitioned into two separate areas by a curtain; the infant and the caregiver sat on one side of the partition, while the experimenter and the research assistant (in some cases) sat on the other side. The infant sat on the caregiver's lap in front of the laptop, which was positioned approximately 40–50 cm away. An ELO13 USB computer speaker, positioned centrally behind the computer screen, was used to present the auditory stimuli. The experimenter observed and recorded the infant's looking behavior using a Logitech C920s Pro HD webcam mounted on the computer screen, facing the infant and the caregiver. The experimenter manually coded the infants’ looking times online during the experiment.

Before each trial, a visual attention‐getter appeared on the screen to attract the infant's attention to the screen. Once the infant fixated on the screen, the experimenter initiated the auditory trial, accompanied by the static, colorful checkerboard that appeared on the screen. Each experiment began with a warm‐up trial to familiarize the infants with the general testing procedure. The auditory stimulus for the warm‐up trial was a musical file (excerpt from a Mozart sonata), immediately followed by 12 test trials, six corresponding to the six lists with the familiar Akan words and six corresponding to the six lists with the pseudowords. Data from the warm‐up trial were not included in the analysis. The 12 test trials were presented in a pseudorandomized order with no more than two trials of the same condition in a row. In blocks of six trials, three familiar words and three pseudoword lists were presented. Each test trial ended after the maximum trial length of 23 s (list duration) was reached or when the infant looked away for more than 2 consecutive seconds. On average, the test session lasted approximately 5 min. Infants’ looking behavior was measured as the duration of time spent looking at the screen while the auditory stimulus was played.

### 2.6 Language Input Assessment

Infants’ language exposure was assessed using the Caregiver Interview about Multilingual Exposure (CIME), as used in previous studies and following the same procedure (e.g., Omane et al. 2024; Omane, Benders, et al. 2025). The CIME was used to estimate the languages directed to the infant and those the infant generally overhears in the environment. Questions were asked about a range of speakers and the languages they speak with the child, as well as a range of regular environments where the child is likely to be exposed to languages. The relative exposure to each language was computed as the proportion of time an infant heard a given language relative to the total time of exposure to all languages across directed and overheard speech (as in Omane et al. 2024; Omane, Benders, et al. 2025).

This study's sample size, design, hypothesis, and analysis were preregistered on Open Science Framework; see: (https://osf.io/m6vp9/overview?view_only=508e2e1500eb4fe18f7fef84e3e08ce9). The data and analysis code will be available in the online repository.

## 3 Data Processing and Analysis

### 3.1 Preregistered Model

The analyses were conducted using R (R Core Team 2023). Analyses of the accumulated looking times on screen in seconds(s) per trial (henceforth LT) as the dependent variable were performed using linear mixed‐effects modeling with the lme4 package (v1.1.33; Bates et al. 2015). A Shapiro–Wilk normality test indicated that the raw data were not normally distributed (W = 0.89, *p* < 0.0001). Therefore, the LT data were log‐transformed and used as the dependent variable in our statistical analysis, as pre‐registered.

The preregistered model included the fixed effects of word type (familiar Akan words vs. pseudowords) coded using a successive difference contrast coding (familiar words coded as –0.5; pseudowords as 0.5), relative exposure to Akan (continuous variable, centered), age in days (continuous variable, centered), and block (first vs. second half of the experiment, contrast coding). Its random structure included random intercepts for participant and list, and a random by‐participant slope of word type. The preregistered model was:

lmer(logLT ∼ word_type * (exposure + age + block) + (1+word_type|participant) + (1|list), data = data).

The preregistered model did not converge and was reduced, as pre‐registered, by removing the random by‐participant slope of word type. The reduced model, which converged, was:

lmer(logLT ∼ word_type * (akan + age + block) + (1| participant) + (1|list), data = data)

## 4 Results and Discussion

Infants’ exposure to Akan, the test language, ranged from 10.01% to 88.7% (mean = 33.4%, SD = 18.3). Other languages of exposure included Ga (14 infants), Ewe (3 infants), Dangme (10 infants), Frafra (2 infants), and Ghanaian English (23 infants). The data showed a total of five languages and eight different language combinations across the sample (Table 3, left panel). Infants had between two and six input providers/caregivers, including parents, grandparents, other relatives, older siblings, and neighbors. Some of these input providers/caregivers spoke more than one language to the infant (Table 4).

**TABLE 3:** Language combinations and number of infants exposed to each combination (*N*) in Experiment 1 (left) and Experiment 2 (right). GhE means Ghanaian English.

Experiment 1		Experiment 2	
Language combinations	*N*	Language combinations	*N*
Akan, GhE	1	Akan, Hausa, GhE	1
Akan, Ga, GhE	9	Akan, Ga, GhE	14
Akan, Dangme, GhE	7	Akan, Dangme, GhE	3
Akan, Dangme, Ga	1	Akan, Ewe, GhE	1
Akan, Ewe, GhE	2	Akan, Dangme, Ga, GhE	2
Akan, Ga, Dangme, GhE	1	Akan, Ewe, Ga, GhE	1
Akan, Ga, Frafra, GhE	2	Akan, Ewe, Dangme, GhE	1
Akan, Ewe, Dangme, Ga, GhE	1	Akan, Ga, Ewe, Krache, GhE	1

**TABLE 4:** Overview of the number of languages spoken to the infant by each caregiver category in Experiment 1, showing the number (and percentage) of caregivers who speak a given number of languages to the infant. Percentages are calculated over the number of caregivers per caregiver category; “total” indicates the total number of caregivers per caregiver category.

	Number of languages spoken to the infant by each caregiver category (retrieved from the CIME)					
Caregiver category	One	Two	Three	Four	Five	Total
Mother	3 (13%)	12 (52%)	6 (26%)	2 (9%)	0 (%)	23
Father	9 (39%)	11 (48%)	3 (13%)	0 (0%)	0 (0%)	23
Grandparents	9 (69%)	3 (23%)	1 (8%)	0 (0%)	0 (0%)	13
Older siblings	5 (38%)	8 (62%)	0 (0%)	0 (0%)	0 (0%)	13
Other relatives	3 (30%)	7 (70%)	0 (0%)	0 (0%)	0 (0%)	10
Other people	6 (75%)	2 (25%)	0 (0%)	0 (0%)	0 (0%)	8
Daycare	0 (0%)	0 (0%)	0 (0%)	0 (0%)	0 (0%)	0
Domestic staff	1 (100%)	0 (0%)	0 (0%)	0 (0%)	0 (0%)	1

**FIGURE 1:**
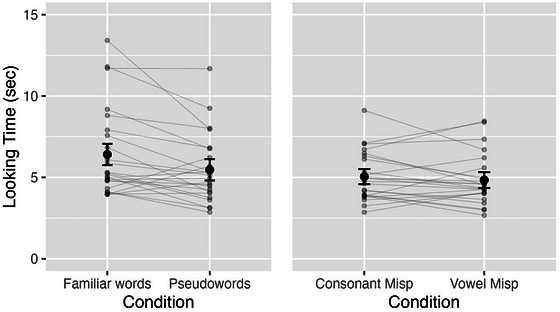
Mean looking times and 95% CIs for familiar words and pseudowords (left panel, Experiment 1) and consonant and vowel mispronunciations (right panel, Experiment 2). Dots indicate individual infants’ means for the two conditions.

The linear mixed‐effects analysis (see model output in Table 5) showed a significant effect of word type (*β* = –0.1701, *p* = 0.03), with infants looking longer during familiar Akan words trials (*M* = 6.4 s, *SD* = 2.7) than to pseudoword trials (*M* = 5.5 s, *SD* = 2.1), indicating a listening preference for familiar Akan words over pseudowords (see Figure 1, left panel). In line with this, 18 of the 24 infants listened longer to the familiar word trials than to pseudoword trials, while only six infants showed the opposite pattern, a significant difference (binomial test, *p *= 0.02).

**TABLE 5:** Final model output of Experiment 1, showing parameters of the Linear Mixed‐Effects Model.

Fixed effects	Estimate *β*	SE	*t* value	*p* value
(Intercept)	1.744	0.083	20.936	<0.0001
Word type	−0.170	0.079	−2.146	0.033
Exposure	0.002	0.004	0.491	0.629
Age	0.003	0.002	1.825	0.083
Block	−0.277	0.056	−4.916	<0.0001
Word type*Exposure	−0.002	0.003	−0.691	0.490
Word type*Age	0.00006	0.002	0.046	0.964
Word type*block	0.106	0.113	0.941	0.347

There was a marginally significant effect of age (*β* = 0.0033, *p* = 0.08), indicating a tendency of older infants to show longer looking times than younger infants in the experiment. However, we found no interaction of age and word type, suggesting no evidence for or against an effect of age on the preference for familiar words. There was a significant effect of block (*β* = –0.2773, *p* < 0.0001), meaning that infants’ LTs to trials decreased over the course of the experiment. There was no significant effect of exposure to Akan (*β* = 0.002, *p* = 0.63), and no interaction was significant (all *p's *> 0.3). The present findings provide the first evidence of wordform recognition in Akan‐learning multilingual infants aged 11–14 months. They extend previous research showing wordform recognition and a familiarity preference in the first year of life in infants learning Indo‐European languages in Europe (e.g., French: Poltrock and Nazzi 2015) to infants learning Akan in a multilingual environment in Africa. We did not find evidence that the word recognition effect was modulated by the relative amount of Akan input infants received, suggesting that the familiarity effect was relatively stable within the large range of Akan exposure (10.01%–88.7%) of our sample.

The present findings also demonstrate the effectiveness of the procedure and design, allowing us to proceed to Experiment 2, which investigates infants’ recognition of consonant and vowel mispronunciations of familiar words using the same methodology, testing whether a V‐bias is found in an African tone language, which would extend the V‐biases found in Asian tone languages. This is the first experiment to investigate the prediction of a V‐bias in lexical processing in Akan‐learning infants, which will also explore whether and how this bias is modulated by the amount of exposure to tone language(s).

### 4.1 Experiment 2: Consonant Versus Vowel Mispronunciations

Experiment 2 investigated 11–14‐month‐old Akan‐learning infants’ preference for consonant versus vowel mispronunciations of the familiar words used in Experiment 1 and whether a one‐feature consonant or vowel change in these words would differentially block recognition. We had two main hypotheses. First, since Akan has lexical tones marked on vowels, we hypothesized that this would give more weight to vowels than consonants in lexical processing in Akan, based on previous investigations of Asian tone languages showing a V‐bias (e.g., Chen et al. 2021). Accordingly, we predicted that Akan‐learning multilingual infants would show a preference for consonant mispronunciations over vowel mispronunciations, because infants at that age prefer listening to familiar over unfamiliar wordforms, and because vowel mispronunciations would hinder wordform recognition to a larger extent, demonstrating a V‐bias. Also, we hypothesized that two factors related to language exposure might modulate the size of infants’ preferences. The first one is percentage exposure to Akan that might positively modulate infants’ preference. From this follows the prediction that if exposure modulates preference, then the V‐bias would be larger in infants who are more exposed to Akan. Second, given that these multilingual infants were also exposed to other tone languages (local languages in Ghana) and to Ghanaian English (the only non‐tone language they hear), it is possible that what modulates the V‐bias is relative exposure to all tone languages versus Ghanaian English. Accordingly, we hypothesized that percentage exposure to all tone languages might positively correlate with the V‐bias, while percentage exposure to Ghanaian English might negatively correlate with the V‐bias. In our statistical model, we included both percentage of Akan and percentage of English (preferring the latter to percentage of all tone languages such that the two measures in the model are independent).

## 5 Method

### 5.1 Participants

Twenty‐four 11‐ to 14‐month‐old infants (14 girls and 10 boys, *M*_age_ = 392 days; range = 336–453) learning Akan and a range of other languages (including Ghanaian English) spoken in Ghana were tested in this study. An additional five infants were tested but excluded from the analyses because of crying. All infants were Africans, born in Ghana and living in urban Accra; they were tested at the same sites as in Experiment 1. As in Experiment 1, the sample size was determined based on prior studies (Hallé and de Boysson‐Bardies 1994; Poltrock and Nazzi 2015), and again we acknowledge that this limited sample may raise potential power concerns, given that this was fieldwork with a limited stay period; 24 infants was the realistic maximum sample we could reach. The procedures for recruiting infants, obtaining consent, and assessing language exposure were identical to those in Experiment 1. Language exposure data showed that infants were exposed to three to five languages (79.1% three languages, 16.7% four languages, and 4.2% five languages). No infant was reported to have any developmental disabilities or delays. Regarding socioeconomic status in terms of maternal education, years of formal education was the same as in Experiment 1. Participants received a fee as compensation for their participation. The study was approved by the Ethics Committee for Humanities at the University of Ghana (Ghana).

### 5.2 Auditory Stimuli

The stimuli consisted of 10 bi‐syllabic CVCV consonant and vowel mispronunciations of the familiar Akan words used in Experiment 1 (see Table 6). To create the consonant and vowel mispronunciations, we changed only one phonological feature in each of the 10 familiar words. For the consonant mispronunciations, we changed either place, manner of articulation or voicing. For the vowel mispronunciations, we changed vowel height, the only one‐feature vowel changes that were possible. For both types of mispronunciations, half of the changes occurred in the first syllable and half in the second syllable. There were no changes in the tonal pattern; thus, the tones remained the same across the two mispronunciation stimuli and the familiar words.

**TABLE 6:** List of familiar words and their mispronounced versions used in Experiment 2, with changed phonemes in bold.

Familiar words	C‐MISP	FC	SC	V‐MISP	FC	SC
tɔ̀fɪ́	tɔ̀sɪ́	Place	2	tʊ̀fɪ́	Height	1
kʷæ̀dú	**g**ʷæ̀dú	Voicing	1	kʷæ̀d**ó**	Height	2
sɔ̀rɪ́	**f**ɔ̀rɪ́	Place	1	s**ʊ̀**rɪ́	Height	1
dʑìnà	dʑì**r**à	Manner	2	dʑ**è**nà	Height	1
wɔ̀fà	**j**ɔ̀fà	Place	1	wɔ̀f**ɛ̀**	Height	2
dʑɥàrɪ̀	dʑɥà**d**ɪ̀	Manner	2	dʑɥ**ɛ̀**rɪ̀	Height	1
pìrà	**t**ìrà	Place	1	pìr**ɛ̀**	Height	2
hùrì	hù**n**ì	Manner	2	h**ò**rì	Height	1
nípá	ní**t**á	Place	2	níp**ɛ́**	Height	2
sɪ̀wá	**f**ɪ̀wá	Place	1	sɪ̀w**ɛ́**	Height	2

Abbreviations: FC, feature changed; MISP, mispronunciation; SC, syllable changed.

The stimuli were recorded by the speaker who recorded the stimuli for Experiment 1, in the same location, with the same recording device and in the same recording session. As with Experiment 1, three tokens of each item were selected, and six pseudo‐randomized lists for each condition (six “consonant mispronunciation lists” and six “vowel mispronunciation lists”) were constructed, using the exact same constraints as in Experiment 1. All lists were 23 s in duration, with an interstimulus interval ranging between 575 and 603 ms. Acoustic analyses were performed on word duration, intensity, and fundamental frequency measures (mean, minimum, and maximum F0) to verify the lack of acoustic differences between the consonant and vowel mispronunciation lists that could have influenced looking times (Table 7).

**TABLE 7:** Acoustic parameters of the stimuli showing the test statistics for the consonant and vowel mispronunciations.

	Conditions		Test statistics
	C‐MISP	V‐MISP	
Duration (ms)	590.8 (63.2)	591.6 (67.2)	*t* (58) = –0.05, *p *= 0.95
Intensity (db)	70.0 (1.7)	70.0 (1.6)	*t* (58) = 0.06, *p *= 0.95
Minimum F0 (Hz)	100.3 (11.5)	107.4 (21.3)	*t* (58) = –1.59, *p *= 0.12
Maximum F0 (Hz)	170.2 (23.5)	167.0 (26.4)	*t* (58) = 0.50, *p *= 0.62
Averages F0 (Hz)	135.5 (15.2)	136.1 (19.0)	*t* (58) = –0.14, *p *= 0.89

Abbreviation: MISP, mispronunciation.

Research hypotheses and methods, including the analysis plan, were pre‐registered (see https://osf.io/hz29j/overview?view_only=93cac24a564049658b74cfdae4556bc5).

### 5.3 Visual Stimuli

The visual stimuli were identical to those in Experiment 1.

### 5.4 Procedure

The experimental and language input assessment procedures were identical to those used in Experiment 1.

## 6 Data Processing and Analysis

### 6.1 Preregistered Model

All statistical analyses were conducted using R (R Core Team 2023). Analyses of the looking time data as the dependent variable were performed using linear Mixed‐Effects modeling with the lme4 package (v1.1.33; Bates et al. 2015). A Shapiro–Wilk test for normality indicated that the raw data were not normally distributed (W = 0.89, *p* < 0.0001). Hence, the raw LT data were log‐transformed and used as the dependent variable in our statistical analysis, as pre‐registered.

The preregistered model included the fixed effects of mispronunciation type (consonant vs. vowel mispronunciation) coded using a successive difference contrast coding (consonant mispronunciations coded as –0.5; vowel mispronunciation as 0.5), relative exposure to Akan, relative exposure to Ghanaian English, and age in days (all continuous variable, centered), and block (first vs. second half of the experiment). Its random structure included random intercepts for participant and list, and a random by‐participant slope of mispronunciation type. The preregistered model was:

lmer(logLT ∼ mispronunciation‐type * (Akan + English + age + block) + (1+misp_type|participant) + (1|list), data = data).

The preregistered model did not converge and was reduced, as pre‐registered, by removing the random by‐participant slope of mispronunciation type and then the random intercept for list since their variances were estimated to be zero. The reduced model, which converged, was:

lmer(logLT ∼ mispronunciation‐type * (Akan + English + age + block) + (1|participant), data = data).

## 7 Results and Discussion

Infants’ exposure to Akan ranged from 5.2% to 56.7% (mean = 22%, SD = 18.3) and Ghanaian English (used as the complement of all tone languages) ranged from 5% to 65% (mean = 20.9%, SD = 14.8). Other languages included Ga (18 infants), Ewe (24 infants), Dangme (6 infants), and Hausa (1 infant). The data showed a total of seven languages and eight different language combinations across the sample (Table 3, right panel). Infants had between two and six input providers/caregivers, including parents, grandparents, other relatives, older siblings, and neighbors (see Table 8). Some of these input providers/caregivers spoke more than one language to the infant.

**TABLE 8:** Overview of the number of languages spoken to the infant by each caregiver category in Experiment 2, showing the number (and percentage) of caregivers who speak a given number of languages to the infant. Percentages are calculated over the number of caregivers per caregiver category; “total” indicates the total number of caregivers per caregiver category.

	Number of languages spoken to the infant by each caregiver category (retrieved from the CIME)					
Caregiver category	One	Two	Three	Four	Five	Total
Mother	3 (13%)	19 (79%)	2 (8%)	0 (0%)	0 (%)	24
Father	12 (52%)	8 (35%)	3 (13%)	0 (0%)	0 (0%)	23
Grandparents	9 (56%)	5 (31%)	2 (13%)	0 (0%)	0 (0%)	16
Older siblings	5 (45.5%)	5 (45.5%)	1 (9%)	0 (0%)	0 (0%)	11
Other relatives	9 (53%)	8 (47%)	0 (0%)	0 (0%)	0 (0%)	17
Other people	4 (36%)	7 (64%)	0 (0%)	0 (0%)	0 (0%)	11
Daycare	1 (100%)	0 (0%)	0 (0%)	0 (0%)	0 (0%)	1
Domestic staff	0 (0%)	0 (0%)	0 (0%)	0 (0%)	0 (0%)	0

The linear mixed‐effects analysis (see model output in Table 9) revealed no significant effect of mispronunciation type (*β* = –0.047, *p* = 0.577), indicating that infants may have paid equal attention to both consonant (*M* = 5.0 s, *SD* = 1.5) and vowel (*M* = 4.8 s, *SD* = 1.6) mispronunciations (Figure 1, right panel). Moreover, 15 (62.5%) of the 24 infants listened longer to consonant than vowel mispronunciations, with 9 (37.5%) infants showing the opposite pattern, a non‐significant difference (binomial test, *p* = 0.31). This result suggests that infants may have equal sensitivity to both types of mispronunciations.

**TABLE 9:** Final model output of Experiment 2, showing parameters of the Linear Mixed‐Effects Model.

Fixed effects	Estimate *β*	SE	*t* value	*p* value
(Intercept)	1.610	0.065	24.944	<0.0001
Word type	−0.047	0.084	−0.558	0.577
Exposure to Akan	−0.001	0.005	−0.247	0.808
Exposure to English	0.005	0.004	1.214	0.239
Age	−0.0002	0.001	−0.137	0.893
Block	−0.364	0.061	−5.985	<0.0001
Word type*Akan	0.0003	0.005	0.055	0.956
Word type*English	0.001	0.004	0.331	0.741
Word type*Age	0.0001	0.002	0.033	0.974
Word type*block	0.037	0.121	0.307	0.759

There was a significant effect of block (*β* = –0.364, *p* < 0.0001), indicating a decrease in infants’ looking time to the trials over the course of the experiment, with longer looks in the first half than in the second half. There were no significant effects of age (*β* = –0.0002, *p* = 0.893), exposure to Akan (*β* = –0.001, *p* = 0.808) and English (*β* = 0.005, *p* = 0.239), and no interaction was significant (all *p*’s > 0.7). The absence of a main effect of age or a significant interaction between age and mispronunciation type suggests that there were no systematic differences in looking behavior in the age range that we investigated (11–14 months). Furthermore, the absence of a mispronunciation effect does not appear to be influenced by the amount of Akan or Ghanaian English exposure.

## 8 General Discussion

The goal of the present study was to explore Akan‐learning multilingual 11‐to‐14‐months infants’ early recognition of correctly pronounced familiar Akan wordforms (Experiment 1) and mispronunciations of these wordforms (Experiment 2), as well as how these abilities are influenced by infants’ relative exposure to Akan (both experiments) and English (Experiment 2). Additionally, the present study examined whether a V‐bias is more generally found in tone languages, extending prior research to an understudied African tone language, Akan, and to multilingual infants learning tone languages alongside a non‐tone language (e.g., Ghanaian English) simultaneously, tested in Accra, Ghana. Using the central fixation procedure, infants’ looking times (LTs) were recorded as they listened to familiar Akan words versus pseudowords (Experiment 1) and consonant‐ and vowel‐mispronunciations of those familiar words (Experiment 2). Relative exposure to Akan and English was assessed using a parental interview assessment questionnaire. Three main findings emerged. First, infants listened longer to familiar Akan words than to pseudowords, indicating recognition of familiar Akan words (Experiment 1). Second, infants attended equally to both the one‐feature consonant mispronunciations and one‐feature vowel mispronunciations, suggesting equal sensitivity to both types of mispronunciations, and failing to provide evidence of either a C‐ or a V‐bias. Third, infants’ relative exposure to Akan (and all tone languages) versus English did not modulate wordform recognition (Experiment 1) and their sensitivity to mispronunciations (Experiment 2). We discuss these findings and future steps.

Regarding wordform recognition, our study shows that Akan‐learning multilingual infants prefer familiar wordforms over pseudowords, demonstrating a familiarity effect in wordform recognition. Our study shows a marginally significant effect of age, with older infants showing longer looking times than younger infants, but no evidence of an interaction between age and preference for familiar words. These results suggest that Akan‐learning multilingual infants’ preference for, and ability to recognize, native‐language familiar wordforms may already be present by 11 months. Moreover, we found no evidence for or against an effect of relative exposure to Akan on infants’ recognition of familiar words, which we will discuss later. Our finding aligns with previous research showing recognition of familiar wordforms over pseudowords/rare words at 11 months across monolingual infants learning different languages: French (Hallé and de Boysson‐Bardies 1994; Poltrock and Nazzi 2015), English (Vihman et al. 2004), and Dutch (Swingley 2005), recently extended to Cantonese (Li et al. Unpublished manuscript). This extends previous findings to a language (Akan) and population (multilingual infants) in Africa not previously described in the literature. Our results provide the first evidence of wordform recognition in Akan‐learning infants and in multilingual infants learning between two and five languages in Ghana.

Regarding the V‐bias, the present study found no preference for consonant or vowel mispronunciations, indicating that infants attend equally to both. We found no effect of age nor an interaction of age and recognition of consonant and vowel mispronunciations, suggesting that performance does not change in the age range tested (11–14 months). Moreover, we found no evidence that infants’ relative attention to consonant and vowel mispronunciations is modulated by relative exposure to Akan and English (used as the complement of all tone languages), as we discuss later. Our findings suggest similar sensitivity to consonant and vowel mispronunciations, failing to establish either a consonant (C‐) or vowel (V‐) bias. Compared to other studies on wordform recognition at 11 months, our result differs from the C‐bias found in French (Poltrock and Nazzi 2015), the V‐bias recently found in Cantonese (Li et al. 2026), but is similar to the null effect found in English (Ratnage et al. 2024).

What factors may have contributed to this lack of bias? First, it is unclear whether the absence of a bias is due to the fact that by 11–14 months, Akan‐learning multilingual infants fail to detect mispronunciations of familiar words, or whether they detect both consonant and vowel mispronunciations to the same degree. If they failed to detect mispronunciations, this would differ from what has been found at the same age in monolingual French and English infants (Hallé and de Boysson‐Bardies 1996; Ratnage et al. 2024). An explanation for this divergent pattern might be linked to their multilingual exposure, implying reduced exposure to Akan (although that factor did not modulate the recognition effect in Experiment 1), but also possible exposure to Akan spoken with a foreign accent by non‐native speakers, leading to phonetically more varied input. If they detected both consonant and vowel mispronunciations to the same degree, this would pattern with findings in English, where 11‐month‐old infants showed no bias, but could detect both consonant and vowel mispronunciations (Ratnage et al. 2024).

The present study does not allow for disentangling between these two possibilities, although as suggested by a reviewer, the looking times for the consonant and vowel mispronunciations in Experiment 2 appear to be shorter than the looking times for the familiar word forms in Experiment 1. Exploratory analyses comparing the two experiments confirm this pattern, showing significant differences between LTs to familiar wordforms and consonant mispronunciations (*t*(42.88) = –2.15, *p* = 0.037, 95% CI [–0.39, –0.01]) and familiar wordforms and vowel mispronunciations (*t*(44.07) = 2.59, *p* = 0.01, 95% CI [0.06, 0.45]), but no significant differences between pseudowords versus consonant mispronunciations (*t*(43.94) = –0.61, *p* = 0.54, 95% CI [–0.24, 0.13]) and pseudowords versus vowel mispronunciations (*t*(44.93) = 1.11, *p* = 0.27, 95% CI [–0.09, 0.30]), according to an independent‐samples Welch's *t*‐test. This suggests longer looking times for familiar wordforms compared to their consonant and vowel mispronunciations, which would be compatible with an ability to detect mispronunciations. However, this interpretation can only be taken as suggestive, given that the comparisons are made across two different groups of infants who heard two different sets of stimuli. Future research will need to directly investigate Akan‐learning infants’ sensitivity to consonant and vowel mispronunciations by directly presenting, within the same experiment, the same infants with familiar wordforms and their consonant and vowel mispronunciations, and compare their looking times to each type.

Second, our findings may indicate a delay in the emergence of a bias in Akan and in multilingual infants at the age at which we tested. Such a delay has been found in other languages, such as English, with the lack of bias at 11 months (Ratnage et al. 2024) being replaced by a C‐bias at 30 months (Nazzi et al. 2009); a V‐bias might thus emerge later in Akan than the 11–14 months age range tested here. Another potential cause of the delayed emergence of a bias could be related to the fact that the infants in our sample were being raised multilingually, with exposure to two to five languages. At 11–14 months of age, these infants may still be trying to figure out where the bias lies. Their difficulty might partly come from the fact that in their environment, all local languages, including Akan, are tone languages for which we would predict a V‐bias, while a C‐bias would be predicted for Ghanaian English. Consistent with this interpretation, the earliest reported evidence of a V‐bias in bilinguals is 24 months in Mandarin–English bilinguals (Wewalaarachchi et al. 2017), an age substantially older than examined here. This suggests that early exposure to multiple languages may delay the emergence of biases in wordform recognition. Further research is needed to determine whether and when Akan‐learning multilingual infants develop such a bias, and to examine how it is shaped by the linguistic environment. Future studies could address these questions by testing older Akan‐learning multilingual infants. Moreover, given the limited research on this topic in bi‐/multilingual infants (see Nazzi 2025, for a review), additional studies are required to better understand how early bi‐/multilingualism affects consonant and vowel processing at the phonolexical level. As apparent from our description of the infants’ linguistic environments (Tables 3, 4, and 8), our sample is characterized by important variation in linguistic input in many factors, including the number and identity of languages, the relative percentage of input of the different languages, and the number of speakers of each language and how many languages they speak to the infant. How these factors affect acquisition will deserve careful evaluation in the future, which will require very large samples and/or more controlled selection of the infants (both constraints that might be out of reach in the context of fieldwork with limited time).

Coming now to the more general goal of the present study, which was to test the proposal that the V‐bias found in previous studies of Asian tone languages (e.g., Wewalaarachchi et al. 2017; Chen et al. 2021) resulted from the fact that these languages have tones, the present study fails to extend evidence of a V‐bias in an African tone language, Akan, failing to bring support to the proposal. However, since this is the first study on Akan and it tests only one age group, our current findings cannot be taken as evidence against this proposal either. This is even more so given that in Asian tone languages, evidence of a V‐bias was not always found: while it was found in Cantonese‐learning 11‐month‐olds (Li et al. 2026) and 30‐month‐olds (Chen et al. 2021) and in both Mandarin monolingual and Mandarin‐English bilingual 24‐month‐olds (Wewalaarachchi et al. 2017;though only in a time course analysis), no bias was found at older ages, whether Mandarin‐English bilingual 2.5‐to‐3.5‐year‐olds and 4‐to‐5‐year‐olds (Singh et al. 2015) or Mandarin monolingual or Mandarin‐English bilingual 6‐year‐olds (Wewalaarachchi and Singh 2020). This suggests that the presence of tones in a language might reduce the relative weight given to consonants, leading to either a V‐bias or a reduced or no C‐bias (as found here for Akan). Differences in linguistic typology may account for the strength of the impact of tones on the bias. Cantonese has a complex tonal system (six tones, both contour and register tones), Mandarin has an intermediate system (four tones, varying in contour), and Akan has a simple system (two register tones). As proposed by Chen et al. (2021), it is thus possible that the more complex the tone system, the stronger the impact on the C‐bias, explaining why a clearer V‐bias was found in Cantonese than in Mandarin, and why we did not find an early V‐bias in Akan. Future studies on Akan should investigate the V‐bias at older ages to determine whether it emerges later in development or, at least, whether no C‐bias is found even in children and adults. Such data is currently lacking, as the present study is the first to address this question. Future research should also investigate whether a V‐bias exists in other African tone languages of different complexity.

Regarding language exposure, the present study finds no evidence for or against an effect of exposure on the recognition of correctly pronounced familiar words (Experiment 1) and their mispronunciations (Experiment 2). In the following, we speculate on possible methodological issues and processing mechanisms in multilingual infants that may have contributed to these null results. First, from a methodological perspective, the absence of an exposure effect may have resulted from the specific familiar words used in our study. These words were selected from the most frequent words in the Akan CDI/checklist (Coffey et al. 2025; Prado et al. 2016) and may therefore occur frequently in infants’ daily input, allowing recognition (and potentially sensitivity to both types of mispronunciations) even with minimal exposure. Consequently, exposure effects may be more evident for less frequent words, which would have to be investigated in future studies. Second, the lack of the Akan exposure effect may be due to the fact that language acquisition, word recognition, and speech perception in Akan‐learning multilingual infants do not depend on the relative amount of input in the target language. Consistent with this interpretation, 6‐month‐old Akan‐learning multilingual infants exposed to one or more ATR‐harmony languages alongside non–vowel harmony languages showed a preference for Akan ATR‐harmonic syllable sequences, with no evidence of exposure effects (Omane et al. 2024). Similarly, Akan‐learning multilingual infants aged 9–11 months segmented speech using ATR harmony cues regardless of relative Akan exposure (Omane, Boll‐Avetisyan, et al. 2025). Our finding is also consistent with previous research on other domains of speech perception, showing no evidence for (or against) the amount of input in a language impacting bilingual infants’ speech segmentation with native language cues (Bosch et al. 2013; Orena and Polka 2019; Polka et al. 2017). Moreover, German/French simultaneous bilingual infants’ preference for trochaic patterns was also found not to be modulated by the amount of exposure to German (Bijeljac‐Babic et al. 2016). It may well be that in bi/multilingual contexts, limited (from 10% to 20%) but regular input with a language provides sufficient information to allow the mechanisms underlying language acquisition to learn basic, salient and frequent phonological and lexical properties at a young age.

In conclusion, our findings show for the first time that multilingual infants, growing up in Ghana, can recognize correctly pronounced familiar words in one of their native languages, Akan, and that this ability is not modulated by their relative exposure to the target language. Additionally, our results suggest that Akan‐learning multilingual infants are equally (in)sensitive to both consonant and vowel mispronunciation. Contrary to our prediction, we found no V‐bias in Akan, despite its being a tone language, but no C‐bias either, which might still attest to a weak effect of tones on the relative weighting of consonants and vowels. Further research on multilingual infants learning Akan and other African tone languages, at different ages and using different methodologies, will be needed to shed more light on the emergence of a C/V bias and how this is modulated by language exposure.

## Author Contributions

**Reginald Akuoko Duah**: resources, supervision, project administration, writing – review and editing, investigation. **Paul Okyere Omane**: conceptualization, investigation, funding acquisition, writing – original draft, writing – review and editing, visualization, methodology, formal analysis, project administration, data curation. **Thierry Nazzi**: conceptualization, funding acquisition, writing – review and editing, methodology, formal analysis, resources, validation, project administration, supervision.

## Ethics Statement

The study was approved by the Ethics Committee for Humanities at the University of Ghana.

## Conflicts of Interest

The authors declare no conflicts of interest.

## Pre‐Registration

This study's design, hypothesis, and analysis were preregistered on the Open Science Framework (see [10.17605/OSF.IO/HZ29J], [10.17605/OSF.IO/M6VP9]).

## Data Availability

The data that support the findings of this study are openly available in OSF at https://doi.org/10.17605/OSF.IO/M4DC5.
